# Dose-dependent artificial prolongation of prothrombin time by interaction between daptomycin and test reagents in patients receiving warfarin: a prospective in vivo clinical study

**DOI:** 10.1186/s12941-017-0203-3

**Published:** 2017-04-11

**Authors:** Makoto Saito, Shuji Hatakeyama, Hideki Hashimoto, Takumitsu Suzuki, Daisuke Jubishi, Makoto Kaneko, Yukio Kume, Takehito Yamamoto, Hiroshi Suzuki, Hiroshi Yotsuyanagi

**Affiliations:** 1 Department of Infectious Diseases, University of Tokyo Hospital, 7-3-1 Hongo, Bunkyo-ku, Tokyo, 113-8655 Japan; 2 Division of General Internal Medicine, Division of Infectious Diseases, Jichi Medical University Hospital, 3311-1 Yakushiji, Shimotsuke-shi, Tochigi 329-0498 Japan; 3 Department of Clinical Laboratory, University of Tokyo Hospital, 7-3-1 Hongo, Bunkyo-ku, Tokyo, 113-8655 Japan; 4 Department of Pharmacy, University of Tokyo Hospital, 7-3-1 Hongo, Bunkyo-ku, Tokyo, 113-8655 Japan

**Keywords:** Daptomycin, Prothrombin time, Warfarin, Drug resistance, Gram-positive infections, High dose

## Abstract

**Background:**

Daptomycin has been reported to cause artificial prolongation of prothrombin time (PT) by interacting with some test reagents of PT. This prolongation was particularly prominent with high concentrations of daptomycin in vitro. However, whether this prolongation is important in clinical settings and the optimal timing to assess PT remain unclear.

**Methods:**

A prospective clinical study was conducted with patients who received daptomycin for confirmed or suspected drug-resistant, gram-positive bacterial infection at a university hospital in Japan. PT at the peak and trough of daptomycin was tested using nine PT reagents. Linear regression analyses were used to examine the difference in daptomycin concentration and the relative change of PT-international normalized ratios (PT-INR).

**Results:**

Thirty-five patients received daptomycin (6 mg/kg). The mean ± standard deviation of the trough and peak concentrations of daptomycin were 13.5 ± 6.3 and 55.1 ± 16.9 μg/mL, respectively. Twelve patients (34%) received warfarin. With five PT reagents, a significant proportion of participants experienced prolongation of PT-INR at the daptomycin peak concentration compared to the PT-INR at the trough, although the mean relative change was less than 10%. None of the participants clinically showed any signs of bleeding. A linear, dose-dependent prolongation of PT was observed for one reagent [unadjusted coefficient β 3.1 × 10^−3^/μg/mL; 95% confidence interval (CI) 2.3 × 10^−5^–6.3 × 10^−3^; p = 0.048]. When patients were stratified based on warfarin use, this significant linear relationship was observed in warfarin users for two PT reagents (adjusted coefficient β, 6.4 × 10^−3^/μg/mL; 95% CI 3.5 × 10^−3^–9.3 × 10^−3^; p < 0.001; and adjusted coefficient β, 8.3 × 10^−3^/μg/mL; 95% CI 4.4 × 10^−3^–1.2 × 10^−2^; p < 0.001). In non-warfarin users, this linear relationship was not observed for any PT reagents.

**Conclusions:**

We found that a higher concentration of daptomycin could lead to artificial prolongation of PT-INR by interacting with some PT reagents. This change may not be clinically negligible, especially in warfarin users receiving a high dose of daptomycin. It may be better to measure PT at the trough rather than at the peak daptomycin concentration.

## Background

Daptomycin is a cyclic lipopeptide antimicrobial agent, which exerts its bactericidal effects against gram-positive bacteria by decreasing the integrity of bacterial phospholipid cell membranes in the presence of Ca^2+^ [1]. It is licensed for use against skin and soft tissue infections and bloodstream infection by gram-positive pathogens. Since its use was first approved in the USA in 2003, several reports have shown that prothrombin time (PT) was prolonged in daptomycin users, especially those who were on warfarin [2, 3]. This prolongation was considered artificial because those patients did not show evidence of bleeding [3]. In addition, daptomycin does not interfere with cytochrome P450 and thus it is unlikely to interact with warfarin in vivo [4]. It is possible that some PT reagents may interfere with daptomycin, resulting in an artificial increase in PT. Indeed, this prolongation was confirmed for some PT reagents in vitro by adding daptomycin to the blood samples, and this effect was particularly prominent in samples with elevated baseline PT due to warfarin use [3, 5, 6]. This finding is critical as a finer control of PT is needed for those treated with warfarin to prevent bleeding and intravascular clotting. In addition, patients who require warfarin and patients who need daptomycin frequently overlap. For example, anticoagulants are used in patients with intravascular devices, who are also at risk for bloodstream infection by gram-positive bacteria. The prolongation effect by daptomycin has been reported to depend on the daptomycin concentration [3, 5, 6], and some have suggested that PT should be measured at the trough rather than at the peak of the daptomycin concentration [3]. However, this suggestion has not been assessed in clinical settings. In addition, the effect of some PT reagents used in Japan on daptomycin has not been evaluated.

In this study, we compared for the first time PT at the trough and the peak concentrations of daptomycin using clinical samples prospectively collected from patients treated with daptomycin. The objective of this study was to assess whether there were any PT reagents affected by daptomycin and whether there was any difference in this effect depending on concomitant use of warfarin.

## Methods

This prospective study was conducted at the University of Tokyo Hospital, Japan between February 2013 and October 2014. Participants were patients treated with daptomycin for confirmed or suspected drug-resistant, gram-positive bacterial infections (i.e. infections caused by methicillin-resistant *Staphylococcus aureus* and vancomycin-resistant enterococci) after they gave full written consents. Children younger than 12 years and anaemic patients with a haemoglobin concentration <9.0 mg/dL were excluded.

We examined the impact of daptomycin on PT measurement by comparing PT-international normalised ratio (PT-INR) at the trough and peak blood concentrations of daptomycin. Daptomycin was administered intravenously at a 6 mg/kg/dose over 30 min every 24 h (clearance of creatinine ≥30 mL/min) or 48 h (<30 mL/min) [4]. Blood samples (one 4.5 mL 3.2% sodium citrate tube for PT and one 2.0 mL ethylenediamine tetraacetic acid anticoagulant tube for daptomycin concentration) were taken at ≤30 min before (trough) and 30–60 min after (peak) the ≥3rd daptomycin administration. Blood samples were also taken for PT measurement at enrolment before the initiation of daptomycin. Within 1 h after collection, plasma was isolated by centrifugation (3000 rpm, room temperature, 10 min; 1500*g*, 4 °C, 15 min) and stored at −80 or −70 °C until assay of PT and daptomycin, respectively. We measured PTs using nine commercial reagents that are commonly used in Japan (Table 1). Samples were examined up to three times for each sample using each reagent, and median values were used for the statistical analyses. Plasma concentrations of daptomycin were measured using ultra-performance liquid chromatography with tandem mass spectrometric detection (UPLC-MS/MS) according to a previous report with some modifications [7]. Briefly, 50 µL of plasma specimens spiked with an internal standard (4-hydroxychalcone, 10 mg/L) was deproteinated by addition of 200 µL of methanol and centrifuged for 15 min at 15,000 rpm, 4 °C. Then, the clear supernatant was diluted 10 times with 80% methanol, and 5 µL aliquots were analysed with a UPLC-MS/MS system consisting of an ACQUITY UPLC^®^ instrument coupled with a Quattro Premier XE triple-quadrupole MS/MS system (Waters Corp., Milford, MA, USA) operated under electrospray ionization (ESI) mode. Chromatographic separation was performed on ACUITY UPLC^®^ BEH C18 column (1.7 µm, 2.1 × 100 mm, Waters Corp.) in isocratic separation mode. The mobile phase was 50/50 (v/v) milliQ water/acetonitrile containing 0.1% formic acid, and the flow rate was set at 0.3 mL/min. Analytes were monitored in multiple reaction monitoring (MRM) mode, and the m/z of precursor and product ions was 811.33 > 313.26 and 223.15 > 117.00 (ESI-) for daptomycin and 4-hydroxychalcone, respectively. The calibration ranges were 0.25–100 mg/L. Samples were anonymised with unique patient identifiers, and laboratories were blinded to the patients’ information.

**Table 1:** Characteristics of prothrombin time test reagents used in the study

No	Brand name	Manufacturer	Laboratory	ISI	Thromboplastin	Phospholipid
1	HemosIL Recombi PlasTin 2G	Instrumental Laboratories	University of Tokyo Hospital	1.01	Recombinant human tissue factor	Synthetic phospholipid
2	Neoplastin plus	Roche Diagnostics	Roche Diagnostics	1.33	Rabbit brain	Confidential
3	STA Neoplastin R	Roche Diagnostics	Roche Diagnostics	0.96	Recombinant human tissue factor	Confidential
4	Dade Innovin	Dade Behring (Sysmex)	Sysmex	1.0	Recombinant human tissue factor	No data
5	Thromborel S	Dade Behring (Sysmex)	Sysmex	1.0	Human placenta	No data
6	Thrombocheck PT	Sysmex	Sysmex	1.6	Rabbit brain	No data
7	Simplastin Excel S	BioMerieux	Kyowa Medex	1.22	Rabbit brain	No PG
8	Simplastin HTF	BioMerieux	Kyowa Medex	1.26	Cultured human lung cell	No PG
9	Coagupia PT–N	Sekisui Medical	Sekisui Medical	1.09	Rabbit brain	Confidential

*ISI* international sensitivity index, *PG* phosphatidylglycerol

Statistical tests were conducted using STATA/MP 14.2 (Stata Corp, College Station, TX, USA). The binomial test and Fisher’s exact test were used for binary and categorical variables, respectively. For continuous variables, t test and linear regression model were used. The Huber-White sandwich estimator was used to measure the standard errors for linear regression analyses. Outliers that lay outside the 95% confidence interval (95% CI) based on the standard error of forecast were excluded from the linear regression model. Effect modification by warfarin use was our a priori interest. More than 10% relative change of PT-INR was regarded as clinically significant [8].

This study was approved by the Ethics Committee of the University of Tokyo Hospital (10026).

## Results

### Patient characteristics

Of the 36 participants recruited for this study, one withdrew because of the cessation of daptomycin before the third administration. Among the remaining 35 patients, 25 (71%) were men, and the median age was 61 years (Table 2). Twelve patients received warfarin. The median baseline PT-INR at trough was 2.62 [interquartile range (IQR) 1.63–3.29] in warfarin users and 1.19 (IQR 1.06–1.42) in non-warfarin users using our routine reagent (Reagent 1). The median time interval between trough and peak blood sample collections was 105 min (IQR 95–120 min). Daptomycin treatment (results determined according to administered dose per body weight and number of administrations before the PT measurement) was comparable between the two groups. Most of the patients were receiving treatment for skin/joint infection (19/35; 54%) or bloodstream infection (12/35; 34%). There were more patients with cardiovascular disease in the warfarin group (8/12; 67%) than in the non-warfarin group (5/23; 22%). A total of 49% (17/35) patients were given concomitant antibiotics in addition to daptomycin. None of the patients showed any signs of bleeding during this study.

**Table 2:** Patient characteristics stratified by warfarin use

Characteristic	All (n = 35)	Warfarin user (n = 12)	Non-warfarin user (n = 23)
Age (years)	61 (51–77)	65.5 (52.5–79)	59 (51–74)
Male	25 (71%)	8 (67%)	17 (74%)
PT-INR at trough	1.39 (1.12–2.38)	2.62 (1.63–3.29)	1.19 (1.06–1.42)
Time interval between trough and peak (min)	105 (95–120)	95 (95–110)	112.5 (90–120)
Daptomycin dose (mg/kg/dose)	6.0 (5.9–6.1)	6.0 (5.9–6.4)	6.0 (5.8–6.0)
Number of daptomycin administered before peak	4 (3–5)	4.5 (4–5.5)	4 (3–5)
Creatinine clearance (mL/min)	84.8 ± 54.1	74.2 ± 40.4	89.9 ± 59.7
Source of infection			
Skin/joint infection	19 (54%)	7 (58%)	12 (52%)
Bacteraemia	12 (34%)	5 (42%)	7 (30%)
Other infections	4 (11%)	0 (0%)	4 (17%)
Comorbidity			
Autoimmune disease	5 (14%)	2 (17%)	3 (13%)
Cancer	6 (17%)	2 (17%)	4 (17%)
Cardiovascular disease	13 (37%)	8 (67%)	5 (22%)
Others	11 (31%)	0 (0%)	11 (48%)
Concomitant antibiotic use	17 (49%)	7 (58%)	10 (43%)

Data are shown as a number (%), mean ± standard deviation or median (interquartile range)

*PT-INR* prothrombin time-international normalised ratio. PT-INR by Reagent 1 is shown

### Comparison of PT-INRs between the trough and the peak concentrations of daptomycin

The average ± standard deviation of the trough and peak concentrations of daptomycin were 13.5 ± 6.3 and 55.1 ± 16.9 μg/mL, respectively. Both trough and peak concentrations were slightly higher in the warfarin group (16.6 ± 6.1, 57.6 ± 16.3) than in the non-warfarin group (11.9 ± 5.9, 53.8 ± 17.5), which could be due to the slightly impaired renal function in the warfarin group. The differences between the trough and peak concentrations were roughly equal (41.0 ± 14.0 in warfarin group and 41.9 ± 14.9 in non-warfarin group). The maximum peak concentration observed in this study cohort was 96.4 μg/mL.

PT-INRs at the trough and the peak daptomycin concentration were compared. Regarding Reagent 1, 2, 3, 7, and 8, more than 70% of patients had higher PT-INR at the peak than at the trough level (Table 3). This trend was not different between warfarin users and non-users for the same PT reagent (data not shown). Based on the PT-INR values, however, no reagents showed a clinically important relative change of >10%. The highest relative change of PT-INR was observed in the warfarin group with Reagent 3 at 1.13 (95% CI 1.03–1.22). The absolute difference of PT-INRs was biggest in warfarin users at 0.26 (95% CI 0.02–0.50) with Reagent 3 and in non-warfarin users at 0.06 (95% CI 0.01–0.12) with Reagent 1.

**Table 3:** Proportion of patients with prolonged PT-INR at peak daptomycin concentration and the relative change of PT-INR between trough and peak

Reagent	PT-INR_peak_ > PT-INR_trough_ (%)	p value^a^	PT-INR_peak_/PT-INR_trough_ (95% CI)	p value^b^
1	26/35 (74%)	0.006	1.05 (1.02–1.07)	1.00
2	27/35 (77%)	0.002	1.04 (1.01–1.07)	1.00
3	31/35 (89%)	<0.001	1.07 (1.03–1.11)	0.94
4	20/35 (57%)	0.50	1.00 (0.98–1.02)	1.00
5	20/35 (57%)	0.50	1.01 (0.98–1.03)	1.00
6	20/35 (57%)	0.50	1.02 (0.97–1.07)	1.00
7	25/35 (71%)	0.02	1.04 (1.01–1.08)	1.00
8	25/35 (71%)	0.02	1.05 (1.01–1.09)	0.99
9	19/35 (54%)	0.74	1.00 (0.98–1.02)	1.00

*PT-INR* prothrombin time-international normalised ratio, *CI* confidence interval

^a^p values by binomial test compared to 0.50

^b^One-sided p value by t test to test whether the ratio is more than 1.10

### Linear relationship between the relative change of PT-INR and the difference in daptomycin concentrations between the trough and peak

**Fig. 1:**
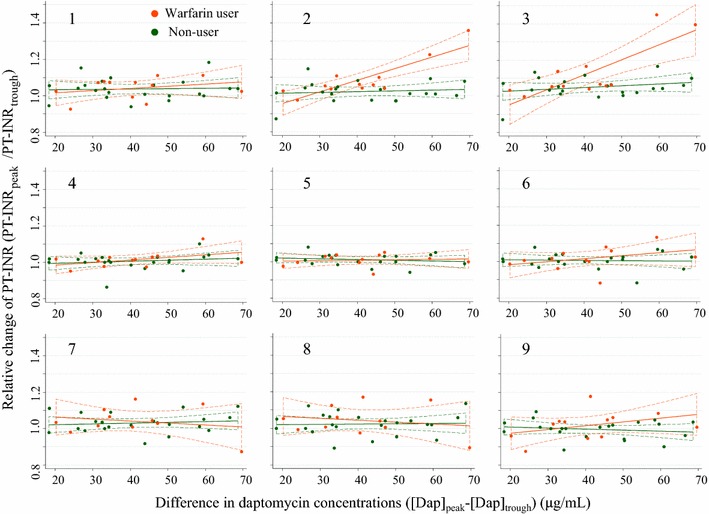
The relative change of PT-INR and the difference in daptomycin concentrations [Dap] between trough and peak. Linear regression lines with a 95% confidence interval for the predicted mean are shown separately for warfarin users and non-users. The *reagent numbers* are shown in the *top left*. *PT-INR* prothrombin time international normalised ratio

The difference in daptomycin concentrations (Δ-daptomycin) between trough and peak was assessed as an explanatory variable for the relative change of PT-INR (Fig. 1). For Reagent 3, a significant linear association was observed between Δ-daptomycin and relative change of PT-INR: unadjusted coefficient β, 3.1 × 10^−3^/μg/mL; 95% CI 2.3 × 10^−5^–6.3 × 10^−3^; p = 0.048; r^2^ = 0.19 (Table 4). This relationship was unchanged after adjusting for warfarin use: adjusted coefficient β, 3.3 × 10^−3^/μg/mL; 95% CI 5.2 × 10^−4^–6.0 × 10^−3^; p = 0.02; r^2^ = 0.34.

**Table 4:** Linear relationship between the relative change of PT-INR and the difference in daptomycin concentrations between the trough and peak

Reagent	n	Unadjusted β (95% CI) (/μg/mL)	p value	r^2^
			H_0_: β = 0	
1	34	5.1 × 10^−4^ (−9.2 × 10^−4^–1.9 × 10^−3^)	0.48	0.02
2	34	2.2 × 10^−3^ (−5.6 × 10^−4^–4.9 × 10^−3^)	0.12	0.15
3	34	3.1 × 10^−3^ (2.3 × 10^−5^–6.3 × 10^−3^)	0.048	0.19
4	34	8.5 × 10^−4^ (−2.2 × 10^−4^–1.9 × 10^−3^)	0.12	0.08
5	32	−1.8 × 10^−4^ (−9.2 × 10^−4^–5.6 × 10^−4^)	0.62	0.006
6	33	4.3 × 10^−4^ (−7.3 × 10^−4^–1.6 × 10^−3^)	0.45	0.02
7	33	−5.0 × 10^−5^ (−2.0 × 10^−3^–1.9 × 10^−3^)	0.96	<0.001
8	34	−2.3 × 10^−4^ (−2.1 × 10^−3^–1.6 × 10^−3^)	0.80	0.003
9	34	2.3 × 10^−4^ (−1.1 × 10^−3^–1.6 × 10^−3^)	0.73	0.003

For Reagent 2 and 3, effect modification by warfarin use was indicated (p value for effect modification, 0.0003 and 0.001, respectively). When taking this effect modification into account, only warfarin users showed a linear association for both Reagent 2 (adjusted coefficient β, 6.4 × 10^−3^/μg/mL; 95% CI 3.5 × 10^−3^–9.3 × 10^−3^; p < 0.001; r^2^ = 0.57) and Reagent 3 (adjusted coefficient β, 8.3 × 10^−3^/μg/mL; 95% CI 4.4 × 10^−3^–1.2 × 10^−2^; p < 0.001; r^2^ = 0.56). No linear association between Δ-daptomycin and relative change of PT-INR was observed for other reagents (Table 5).

**Table 5:** Adjusted linear relationship between the relative change of PT-INR and the difference in daptomycin concentrations between the trough and peak

Reagent	Adjusted β (95% CI) (/μg/mL)	p value	p value for interaction	r^2^
		H_0_: β = 0		
1	5.1 × 10^−4^ (−9.5 × 10^−4^–2.0 × 10^−3^)	0.48		0.02
Warfarin (+)	1.2 × 10^−3^ (−1.3 × 10^−3^–3.7 × 10^−3^)	0.34	0.79	0.03
Warfarin (−)	2.0 × 10^−4^ (−1.7 × 10^−3^–2.1 × 10^−3^)	0.83		
2	2.3 × 10^−3^ (−1.1 × 10^−4^–5.0 × 10^−3^)	0.06		0.33
Warfarin (+)	6.4 × 10^−3^ (3.5 × 10^−3^–9.3 × 10^−3^)	<0.001	0.0003	0.57
Warfarin (−)	4.2 × 10^−4^ (−1.6 × 10^−3^–2.5 × 10^−3^)	0.68		
3	3.3 × 10^−3^ (5.2 × 10^−4^–6.0 × 10^−3^)	0.02		0.34
Warfarin (+)	8.3 × 10^−3^ (4.4 × 10^−3^–1.2 × 10^−2^)	<0.001	0.001	0.56
Warfarin (−)	9.8 × 10^−4^ (−1.2 × 10^−3^–3.2 × 10^−3^)	0.36		
4	8.5 × 10^−4^ (−2.5 × 10^−4^–2.0 × 10^−3^)	0.13		0.08
Warfarin (+)	1.4 × 10^−3^ (−1.1 × 10^−3^–3.9 × 10^−3^)	0.26	0.52	0.10
Warfarin (−)	5.6 × 10^−4^ (−6.1 × 10^−4^–1.7 × 10^−3^)	0.33		
5	−1.8 × 10^−4^ (−9.2 × 10^−4^–5.7 × 10^−4^)	0.63		0.009
Warfarin (+)	2.3 × 10^−4^ (−7.1 × 10^−4^–1.2 × 10^−3^)	0.63	0.78	0.03
Warfarin (−)	−4.0 × 10^−4^ (−1.4 × 10^−3^–6.5 × 10^−4^)	0.44		
6	4.4 × 10^−4^ (−7.3 × 10^−4^–1.6 × 10^−3^)	0.45		0.03
Warfarin (+)	1.7 × 10^−3^ (−4.7 × 10^−4^–3.8 × 10^−3^)	0.12	0.45	0.09
Warfarin (−)	−1.4 × 10^−4^ (−1.5 × 10^−3^–1.2 × 10^−3^)	0.84		
7	−3.3 × 10^−5^ (−2.1 × 10^−3^–2.0 × 10^−3^)	0.97		0.006
Warfarin (+)	−1.1 × 10^−3^ (−5.9 × 10^−3^–3.8 × 10^−3^)	0.65	0.81	0.03
Warfarin (−)	4.4 × 10^−4^ (−1.3 × 10^−3^–2.2 × 10^−3^)	0.61		
8	−2.1 × 10^−4^ (−2.2 × 10^−3^–1.8 × 10^−3^)	0.83		0.02
Warfarin (+)	−1.0 × 10^−3^ (−5.6 × 10^−3^–3.5 × 10^−3^)	0.65	0.72	0.04
Warfarin (−)	1.5 × 10^−4^ (−1.7 × 10^−3^–2.0 × 10^−3^)	0.87		
9	2.6 × 10^−4^ (−1.1 × 10^−3^–1.7 × 10^−3^)	0.71		0.03
Warfarin (+)	2.1 × 10^−3^ (−7.9 × 10^−4^–4.9 × 10^−3^)	0.15	0.32	0.12
Warfarin (−)	−5.6 × 10^−4^ (−2.1 × 10^−3^–9.6 × 10^−4^)	0.46		

For each PT reagent, the first row is based on the linear regression model adjusted for warfarin use, and the second and the third rows are strata-specific values based on the linear regression model with interaction by warfarin use

## Discussion

PT-INR at the peak concentration increased compared to that at the trough concentration of daptomycin in 56% (5/9) of the reagents, although the difference was not clinically meaningful (<10%). The magnitude of PT prolongation, however, could depend on the concentration of daptomycin. Based on the linear regression results in warfarin users, the PT-INR at a daptomycin peak concentration of 70 μg/mL is predicted to be 1.15 times (Reagent 2) and 1.20 times (Reagent 3) higher than the PT-INR at a trough concentration of 20 μg/mL. These estimates are in the similar range of effects previously reported by in vitro studies (ranging from 1.15 to more than three times higher at 100 μg/mL than at 0 μg/mL) [3, 5, 6, 9]. These magnitudes of elevation are clinically relevant, especially considering that higher daptomycin dosing (>6 mg/kg) was recently suggested and used for certain clinical situations [10, 11].

Susceptibility of PT reagents to the interaction with daptomycin has been reported to depend on two factors: the type of thromboplastin reagents used and the condition of phospholipids [3, 5]. One common feature among the affected reagents was that they were derived from recombinant human or rabbit tissue factors [3, 5, 6]. Phosphatidylglycerol (PG) concentration is considered to be the other key factor for this interaction [5]. When PG was added, reagents containing recombinant rabbit or recombinant human tissue factor showed concentration-dependent prolongation of PT by daptomycin, whereas a reagent containing human placenta was less affected [5]. One of the two affected reagents in our study contained recombinant human tissue factor, whereas the other contained rabbit brain. All samples were centrifuged within 1 h after collection and then kept at −80 °C; therefore, the time between collection and measurement, and transportation were not likely to considerably affect our results [12].

Our comparison between trough and peak concentrations was based on the assumption that PT was otherwise unchanged between trough time and peak time, which were 1–3 h apart on the same day. Although factors other than increased daptomycin concentration might have caused true prolongation of PT between the trough and peak concentrations, such factors were not likely, and even if they played a role in the effect, they would not explain why this prolongation was only observed for the two PT reagents. First, this trend was not attributable to increased warfarin concentration, as only one patient took warfarin between the two sample collections, and all others took warfarin after blood collection at peak of daptomycin. Second, the circadian rhythm of PT, which is approximately 5–10% [13–15], did not likely to affect our results. If this circadian rhythm was affecting our results, all reagents should have shown the same pattern. Furthermore, although the PT seems to vary in a day, it is still controversial whether there is a certain circadian pattern of PT that is the same across patients on warfarin [13–15]. Third, the impact of the concomitant use of other antibiotics on the change of PT between the two time points could be limited. There are mainly three mechanisms by which antibiotics can affect PT: interaction between warfarin and antibiotics; reduced vitamin K production resulting from interference by the N-methylthiotetrazole side chain of certain antibiotics; and the effect of antibiotics on normal gut flora producing vitamin K. Only one patient took both warfarin and a concomitant antibiotic that was known to interact with warfarin. This patient took sulphamethoxazole-trimethoprim (ST) and he was the only person in the warfarin group who showed more than a 5% relative increase in PT-INR with all PT reagents. Nonetheless, Reagent 2 and 3 showed a greater relative increase in PT-INR (1.23 and 1.45, respectively) than the rest of the PT reagents (mean 1.11, 95% CI 1.07–1.15). Interaction between ST and test reagents was also unlikely, as this prolongation of PT by Reagent 2 and 3 was not observed in the other three patients who used ST. There was only one patient who took warfarin and an antibiotic with a N-methylthiotetrazole side chain (i.e. cefmetazole). Similar to the previous patient, only Reagents 2 and 3 showed an increase in PT-INR in this patient (relative increase of 1.36 and 1.40, respectively), and the mean value for the rest of the PT reagents did not increase (mean 0.98, 95% CI 0.92–1.03). Thus, true prolongation of PT by the N-methylthiotetrazole side chain of cefmetazole was unlikely. Additionally, artificial prolongation of PT resulting from the interaction between cefmetazole and the two PT test reagents was unlikely. This was because the blood level of cefmetazole was considered to decrease between the two time points of blood sample collection, as cefmetazole was administered 3 h before the first blood sample collection. The effect on normal gut flora was not likely to change PT dramatically in 1–3 h. Lastly, the prolongation of PT was not likely due to regression to the mean, as this trend of PT prolongation was unchanged even when patients with a lower initial PT-INR of <1.0 were excluded from the analyses (data not shown).

There were some differences between warfarin users and non-users. Both the absolute and relative increases in PT-INR between trough and peak were smaller in non-warfarin users compared to those in warfarin users. Similarly, the dose-dependent effect of daptomycin on PT prolongation was only observed in warfarin users in our study. In previous in vitro studies, normal plasma samples were less reactive to daptomycin than samples from patients with anti-vitamin K therapy [5], or with prolonged baseline PT (PT-INR > 2.0) [6]. Another study revealed that samples with normal PT were slightly less affected by daptomycin than that those obtained from warfarin users, whereas warfarin users with different PT levels showed a similar increase rate [3]. Another possibility is that the apparent discrepancy between the response in warfarin users and the non-response in non-users in our study might be due to the relatively low peak concentration of daptomycin in our patients rather than the warfarin effect. In all in vitro studies, changes of PT in normal PT samples were small, particularly if the daptomycin concentration was low.

This study has some limitations. As we used clinical samples, we could not finely control the daptomycin concentration, which led to a relatively small difference between trough and peak daptomycin concentrations. Samples with a high peak concentration (e.g. >70 μg/mL) were also scarce in our study. Therefore, care is required when interpreting our results showing an increasing trend in the relative change of PT-INR caused by increased difference in the daptomycin concentrations for Reagents 2 and 3 because the prediction relied on a small number of samples with a high peak concentration. It is also possible that other tested agents are affected by daptomycin under a higher peak concentration. We cannot conclude whether the effect of daptomycin occurred in warfarin users or patients with high PT-INR for any reason because these patients largely overlapped in this study. Therefore, in the future, it is necessary to assess whether the measurement of PT from patients with elevated PT due to coagulopathy but not on warfarin will be affected by daptomycin.

## Conclusion

In summary, we found that a higher concentration of daptomycin could lead to artificial prolongation of PT-INR by interacting with some PT reagents, particularly in patients on warfarin. Because we used clinical samples, the results of this study relied on a relatively small number of samples, especially those with a high peak daptomycin concentration, which could have been influenced by many time-varying confounding factors in addition daptomycin concentration. Therefore, in vitro studies assessing the impact of daptomycin on some reagents may be warranted. In the meantime, we suggest that it may be better to measure PT near the trough concentration of daptomycin, especially when PT is elevated or warfarin is used.

## Abbreviations

CI: confidence interval
ESI: electrospray ionization
IQR: interquartile range
MRM: multiple reaction monitoring
PG: phosphatidylglycerol
PT: prothrombin time
PT-INR: prothrombin time international normalised ratios
UPLC-MS/MS: ultra-performance liquid chromatography with tandem mass spectrometric detection
USA: United States of America

## References

[1] Steenbergen JN, Alder J, Thorne GM, Tally FP (2005). Daptomycin: a lipopeptide antibiotic for the treatment of serious Gram-positive infections. J Antimicrob Chemother.

[2] Drug information: CUBICIN^®^ (daptomycin for injection). 2016. https://www.merck.com/product/usa/pi_circulars/c/cubicin/cubicin_pi.pdf. Accessed 25 Oct 2016.

[3] Webster PS, Oleson FB, Paterson DL, Arkin CF, Mangili A, Craven DE, Adcock DM, Lindfield KC, Knapp AG, Martone WJ (2008). Interaction of daptomycin with two recombinant thromboplastin reagents leads to falsely prolonged patient prothrombin time/International Normalized Ratio results. Blood Coagul Fibrinolysis.

[4] Kosmidis C, Levine DP (2010). Daptomycin: pharmacology and clinical use. Expert Opin Pharmacother.

[5] van den Besselaar AM, Breukink E, Koorengevel MC (2010). Phosphatidylglycerol and daptomycin synergistically inhibit tissue factor-induced coagulation in the prothrombin time test. J Thromb Haemost.

[6] Yamada T, Kato R, Oda K, Tanaka H, Suzuki K, Ijiri Y, Ikemoto T, Nishihara M, Hayashi T, Tanaka K (2016). False prolongation of prothrombin time in the presence of a high blood concentration of daptomycin. Basic Clin Pharmacol Toxicol.

[7] Verdier MC, Bentue-Ferrer D, Tribut O, Collet N, Revest M, Bellissant E (2011). Determination of daptomycin in human plasma by liquid chromatography-tandem mass spectrometry. Clinical application. Clin Chem Lab Med.

[8] Poller L (1998). Screening INR deviation of local prothrombin time systems. J Clin Pathol.

[9] van den Besselaar AM, Tripodi A (2007). Effect of daptomycin on prothrombin time and the requirement for outlier exclusion in International Sensitivity Index calibration of thromboplastin. J Thromb Haemost.

[10] Baddour LM, Wilson WR, Bayer AS, Fowler VG, Tleyjeh IM, Rybak MJ, Barsic B, Lockhart PB, Gewitz MH, Levison ME (2015). Infective endocarditis in adults: diagnosis, antimicrobial therapy, and management of complications: a Scientific Statement for Healthcare Professionals From the American Heart Association. Circulation.

[11] Seaton RA, Gonzalez-Ruiz A, Cleveland KO, Couch KA, Pathan R, Hamed K (2016). Real-world daptomycin use across wide geographical regions: results from a pooled analysis of CORE and EU-CORE. Ann Clin Microbiol Antimicrob.

[12] van Geest-Daalderop JH, Mulder AB (2005). Boonman-de Winter LJ, Hoekstra MM, van den Besselaar AM: Preanalytical variables and off-site blood collection: influences on the results of the prothrombin time/international normalized ratio test and implications for monitoring of oral anticoagulant therapy. Clin Chem.

[13] Bleske BE, Welage LS, Warren EW, Brown MB, Shea MJ (1995). Variations in prothrombin time and international normalized ratio over 24 hours in warfarin-treated patients. Pharmacotherapy.

[14] García A, Marín F, Sánchez B, Roldán V, Marco P (2002). Diurnal variation in the intensity of anticoagulation in atrial fibrillation. Stroke.

[15] Ho C-H, Lin M-W, You J-Y, Chen C-C, Yu T-J (2002). Variations of prothrombin time and international normalized ratio in patients treated with warfarin. Thromb Res.

